# Sea Sickness

**Published:** 1881-12

**Authors:** 


*Sea Sickness.*—A Practical Treatise on its Symptoms, Nature, and Treatment. By Geo. M. Beard, A. M., M. D. Publisher: E. B. Treat, New York.

The Author having had much experience at sea treats his subject in an interesting and practical manner on the basis of its being a functional disease of the central nervous system.

